# Feasibility and Acceptability of Mindfulness-Based Group Visits for Smoking Cessation in Low-Socioeconomic Status and Minority Smokers with Cancer

**DOI:** 10.1089/acm.2019.0016

**Published:** 2019-07-16

**Authors:** Marjory Charlot, Salvatore D'Amico, Man Luo, Ahmed Gemei, Hasmeena Kathuria, Paula Gardiner

**Affiliations:** ^1^ University of North Carolina Lineberger Comprehensive Cancer Center, Chapel Hill, NC.; ^2^ Boston Medical Center, Boston, MA.; ^3^ The Pulmonary Center, Boston University School of Medicine, Boston, MA.; ^4^ Department of Family Medicine, University of Massachusetts Medical School, Worcester, MA.

**Keywords:** mindfulness, smoking, cancer, medical group visits, health disparities

## Abstract

**Objective:**

Smoking cessation studies tailored for low-income and racial/ethnic minority cancer patients are limited. African American and low-socioeconomic status (SES) smokers have higher cancer mortality rates and are less likely to use evidence-based smoking cessation treatments compared with white and higher SES counterparts. Mindfulness training is a promising approach to address racial and SES disparities in smoking cessation. The authors assessed the feasibility and acceptability of a mindfulness-based smoking cessation (MBSC) medical group visit for low-income and racially diverse smokers with cancer.

**Design and intervention:**

The authors adapted the integrative medical group visit model used for chronic pain and included the You Can Quit smoking cessation curriculum used at the study site, Tobacco Treatment Center. The program was conducted in eight weekly 2-h visits. The authors then tested the feasibility and acceptability of this intervention for actively smoking cancer patients and cancer survivors in two pilot groups (*N* = 18) using a pre–post design.

**Setting/Location:**

This study took place at Boston Medical Center, a large urban safety net academic teaching hospital.

**Outcome measures:**

The authors used a medical group visit intake form to collect data on weekly cigarette intake and home practice. They also gathered additional qualitative data from focus groups and in-depth interviews.

**Results:**

Over 50% of participants (*n* = 10) self-identified as black and 56% reported an annual income of $20,000 or less. Over two-thirds of the participants attended four or more of the eight group visits. There was a significant decrease in weekly cigarette intake from 75.1 cigarettes at baseline to 44.3 at 3 months (*p* = 0.039). None of the participants quit smoking. Participants were satisfied with the program and reported positive lifestyle changes.

**Conclusion:**

MBSC group visits are feasible and acceptable among racially diverse and low-SES smokers with cancer and should be further studied in a larger cohort.

## Introduction

Continued smoking after a cancer diagnosis negatively impacts cancer treatment effectiveness, quality of life, and survival.^1–3^ Smoking cessation after a cancer diagnosis reduces cancer mortality in both smoking- and nonsmoking-related cancers.^4–7^ In 2015, ∼12% of the estimated 15 million adult cancer survivors in the United States were current cigarette smokers,^8,9^ but data from the National Health Information Survey demonstrated that only 51% of patients with cancer who smoke are advised to quit.^10^ Racial/ethnic minority and low-socioeconomic status (SES) populations with cancer are less likely to quit smoking after a cancer diagnosis and have higher cancer mortality rates compared with their white and higher SES counterparts.^11,12^

Interventions addressing racial and SES disparities in smoking cessation among cancer patients are limited. Published studies on smoking cessation in cancer populations include mostly white participants.^13–15^ Despite current practice guidelines for behavioral interventions combined with pharmacotherapy for smoking cessation, low-SES and minority smokers are less likely to use these methods due to limited access to these treatments, cost, and misconceptions about the efficacy and safety of pharmacotherapy.^16–18^ Targeted interventions to improve smoking cessation success in low-SES and minority cancer populations are critical to reducing racial disparities in cancer outcomes.

One potential strategy to address racial and socioeconomic disparities in smoking cessation among cancer patients is mindfulness training. The practice of mindfulness meditation, defined as nonjudgmental moment-to-moment awareness, in clinical settings is based on Jon Kabat-Zinn's Mindfulness-Based Stress Reduction Program.^19^ This method is used to engage individuals in mindfulness meditation and mindfulness yoga for health enhancement and stress reduction. Mindfulness training is a promising nonpharmacologic approach shown to help with smoking cessation.^20^ In a study of 399 African American smokers, high dispositional mindfulness, defined as the tendency for mindfulness attention in daily life, was associated with higher likelihood of quitting smoking.^21^ Moreover, another study demonstrated the feasibility of a mindfulness-based intervention for self-management of anxiety and depression in cancer patients with metastatic breast cancer.^22^ To the authors' knowledge, the use of mindfulness training for smoking cessation in cancer patients has not been reported. The aim of this study was to assess the feasibility and acceptability of a mindfulness-based intervention to engage low-SES and minority cancer patients in evidence-based smoking cessation treatment.

## Methods

### Recruitment and enrollment

Participants were recruited from Boston Medical Center, a large, urban safety net hospital in New England. Recruitment occurred in the outpatient oncology clinic and cancer support groups. Study flyers were posted in the oncology clinic waiting room, intake area, examination rooms, and the infusion center. Medical assistants from the oncology clinic assisted with identifying potential study participants. The standard routine practice of the medical assistants was to record the smoking status for every patient at every clinic visit in the electronic medical record. The medical assistants were asked to offer a study fact sheet to all identified current smokers and to maintain a list of patients interested in being contacted by the study research team. Providers could also inform their patients of the study and notify the research assistant through the electronic medical record with names of patients interested in the study. To assess eligibility, the research assistant contacted potential participants by phone and obtained verbal consent to review the electronic medical record. All eligible patients interested in the study met with the research assistant to review and sign a written informed consent for study participation.

### Study design and intervention

A single-arm observational cohort study design was used to test the feasibility and acceptability of a mindfulness-based smoking cessation (MBSC) medical group visit for low-income and minority smokers with cancer. Patients with a history of cancer, older than 18 years of age, access to a telephone, any cigarette intake within the past week, and the ability to provide informed consent and answer survey questions in English were eligible to participate in the study. Patients were excluded if they were actively using evidence-based smoking cessation treatment, had a cancer prognosis less than 6 months, had untreated mental illness, or were pregnant.

The MBSC intervention was an 8-week 2-h medical group visit cofacilitated by a clinician (M.C. and P.G.) and a certified Mindfulness-Based Stress Reduction trainer (B.J.). Tobacco treatment specialists (a nurse practitioner from the Boston Medical Center Tobacco Treatment Center and a pharmacist from the Hematology/Oncology clinic) were guest facilitators. Two cohorts participated in the 8-week program (Table 1). The MBSC curriculum consisted of two main components: a low-literacy-adapted mindfulness training program tailored by and for low-SES minority patients with chronic back pain^23^ and the You Can Quit smoking cessation curriculum adapted from the Boston Medical Center Tobacco Treatment Center (Table 2).

**Table 1.** Mindfulness-Based Smoking Cessation Program Participant Characteristics

	*Total (*n* = 18)*	*Cohort 1 (*n* = 8)*	*Cohort 2 (*n* = 10)*
Age (50–70), years, mean (SD)	64.2 (8.0)	63.0 (6.6)	65.1 (9.1)
Gender, *n* (%)			
Female	11 (61.1)	5 (62.5)	6 (60.0)
Male	7 (38.9)	3 (37.5)	4 (40.0)
Race, *n* (%)			
Non-Hispanic white	8 (44.4)	3 (37.5)	5 (50.0)
Non-Hispanic black	10 (55.6)	5 (62.5)	5 (50.0)
Primary language, *n* (%)			
English	17 (94.4)	7 (87.5)	10 (100)
Other	1 (5.6)	1 (12.5)	0 (0)
Education level, *n* (%)			
High school or less	8 (44.4)	1 (12.5)	7 (70.0)
More than high school	10 (55.6)	7 (87.5)	3 (30.0)
Working status, *n* (%)			
Working	4 (22.2)	2 (25.0)	2 (20.0)
Not working	14 (78.8)	6 (75.0)	8 (80.0)
Income, *n* (%)			
<$10K	5 (27.8)	3 (37.5)	2 (20.0)
$10K–$19.99K	5 (27.8)	4 (50.0)	1 (10.0)
$20K–$49.99K	4 (22.2)	0 (0)	4 (40.0)
Refused/DK	4 (22.2)	1 (12.5)	3 (30.0)
Type of cancer, *n* (%)			
Lung	10 (45.5)	3 (30.0)	7 (58.3)
Breast	4 (18.2)	1 (10.0)	3 (25.0)
Colon	2 (9.1)	1 (10.0)	1 (8.3)
Prostate	2 (9.1)	1 (10.0)	1 (8.3)
Leukemia	1 (4.6)	1 (10.0)	0 (0)
Melanoma/skin cancer	1 (4.6)	0 (0)	1 (8.3)
Other^a^	5 (22.7)	3 (30.0)	2 (16.7)
Active cancer treatment, *n* (%)			
Yes	8 (44.4)	6 (75.0)	2 (20.0)
No	10 (55.6)	2 (25.0)	8 (80.0)
Cigarettes smoked each day, *n* (%)			
10 or less	10 (55.6)	5 (62.5)	5 (50.0)
11–20	8 (44.4)	3 (37.5)	5 (50.0)

^a^ Other types of cancers: chronic lymphocytic leukemia, liver, rectal, vulvar, and endometrial cancers.

DK, don't know; SD, standard deviation.

**Table 2.** Mindfulness-Based Smoking Cessation Curriculum

*Title*	*Clinical discussions*	*Experiential activities*
Session 1	Group orientation	AOB meditation, confidentiality agreement, guidelines for ground rules, introduction to mindfulness, and orientation to weekly assessment
Session 2	You Can Quit presentation led by tobacco treatment specialists	Nine-dot exercise, upstream–downstream parable, and body scan
Session 3	Our bodies and smoking	SMART goals, raisin-eating exercise, mind–body connection, and pleasant event chart
Session 4	Our bodies' response to cravings and withdrawals	Mindful chair yoga sequence, mind–body approaches to craving and withdrawals, and unpleasant event chart
Session 5	Medications for smoking cessation	Opening AOB meditation and walking meditation
Session 6	Stress and anxiety	Opening AOB meditation, gratitude journaling, and awareness meditation
Session 7	Approaches to anxiety and challenging communications	Opening AOB meditation, STOP exercise, challenging communications chart, and loving kindness meditation
Session 8	Wellness review	Opening AOB meditation, self-care massage, SMART goals, and fence visualization meditation

AOB, awareness of breath; SMART, specific, measurable, attainable, relevant, and timely; STOP, stop, take, observe, and proceed.

The mindfulness-based group visits consisted of guided instruction in mind–body practices, such as mindful eating, sitting, and walking meditation, gentle stretching, self-massage, and chair yoga. Each participant received audio compact discs on meditation, body scan, and chair yoga (each 20 min in length), a compact disc player for those who did not own one, and a program manual to facilitate home practice. At the end of each session, participants were encouraged to complete home practice assignments found in the program manual to further engage in the mind–body exercise practiced during the session. The tobacco treatment component of the program consisted of lessons on the effects of smoking and smoking cessation on the body, resources for additional smoking cessation counseling, and pharmacotherapy recommendations, which were referred back to participant's primary care or oncology provider. Each medical group visit started with completion of a clinical intake form, followed by check-in with the clinician. After the check-in, the group visit opened with a centering meditation, followed by an opportunity for participants to describe their experience in the previous week with smoking, attempts to quit smoking, craving and withdrawal symptoms, meditation, and other mind–body practices detailed in the home practice assignment. Following this, participants engaged in an interactive discussion of a health topic related to smoking or smoking cessation. After the discussion, participants were given a 10–15-min break and the remainder of the group visit was spent on guided instruction in mind–body practices, as outlined in Table 2.

All participants signed a confidentiality agreement before the start of the intervention to approve the discussion of protected health information in the group setting and to respect the confidentiality of others in the group by not disclosing any participant information outside of the group visit.

Following completion of each MBSC intervention, the authors conducted a focus group and invited all enrolled participants to attend. Patient interview guides were developed using the modified grounded theory to assess facilitators and barriers to participation in the program, satisfaction with the program, and curriculum content.^24^ Both focus groups were conducted in person at the outpatient oncology clinic. A trained qualitative interviewer (A.L.) obtained informed consent from participants immediately before initiating the interview/focus group. Sessions were audio-recorded and professionally transcribed.

Participants earned up to $95 for participation in the program ($20 for completing the baseline survey, $25 for participating in the group visits, $25 for the focus group, and $25 for the 3-month follow-up).

### Outcome measures

Responses from the focus group, in addition to weekly attendance log and documentation of any home practice on the clinical intake form, were used to assess the feasibility and acceptability of the MBSC program. Feasibility was measured by the ability to recruit and enroll 8–10 participants for each of the 8-week programs, individual participant attendance in 4 or more of the 8 group visits, and feedback during the focus groups on retention and adherence. Acceptability was measured by feedback on the group visit model, curriculum, and lifestyle changes reported by participants during the focus groups. Seven-day cigarette intake was used to assess decrease in cigarette intake as well as smoking cessation measured at baseline, weekly during the intervention, and at the 3-month follow-up.

### Data analyses

A mixed method approach was employed to assess feasibility and acceptability of the intervention for smoking cessation. For quantitative analyses, demographic data were summarized with descriptive statistics, including means, medians, and standard deviations (SDs) for continuous variables and frequencies and percentages for categorical variables. Outcome measures at baseline, at the end of the intervention at 8 weeks, and at 20 weeks (3-month follow-up) were compared using the Wilcoxon rank test for nonparametric variables and the one-sample *t*-test was used for parametric continuous variables. All quantitative analyses were conducted using SAS, version 9.3.^25^

For qualitative analyses, two research assistants trained in qualitative methodology independently coded the transcripts using NVivo 10 qualitative data analysis software.^26^ *A priori* codes were derived from the focus group moderator guide and a thematic analysis approach was used to generate new codes after transcript review.^27^ The coding team members independently merged the codes and resolved any differences in codes by consensus and transcript review.

This study was approved by the Boston Medical Center Institutional Review Board and registered at Clinicaltrials.gov on June 10, 2016, Protocol No. NCT02795312.

## Results

### Recruitment and enrollment

**FIG. 1.**
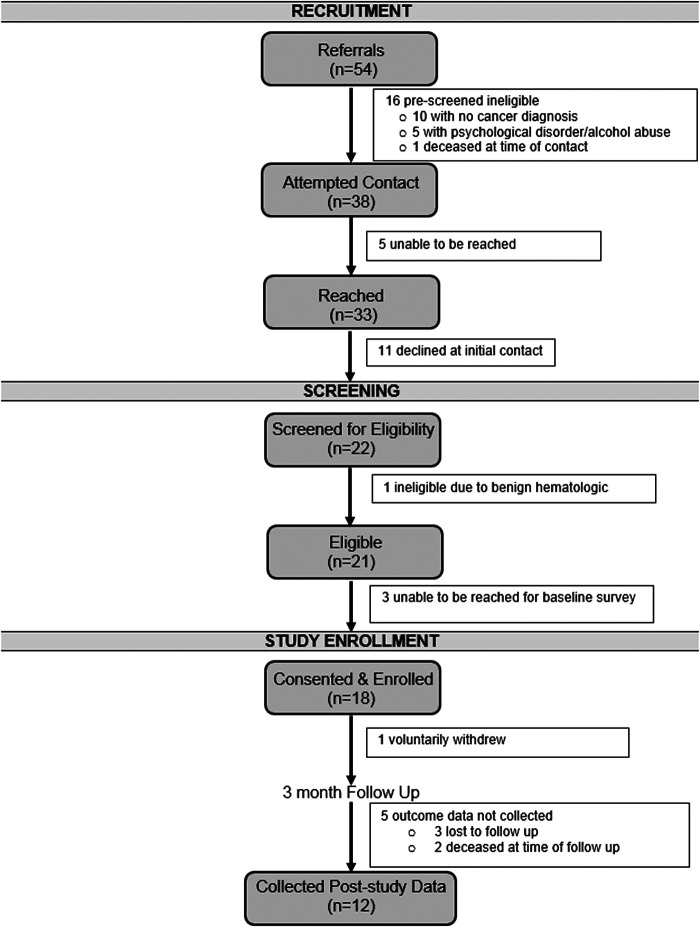
Mindfulness-based smoking cessation program consort diagram.

A total of 54 referrals were made to the MBSC program. Of those, 33 patients were reached and 22 patients agreed to be screened for eligibility. Of the 21 eligible patients, 18 were consented and enrolled (Fig. 1). The MBSC program consisted of 2 cohorts with 8 and 10 participants in the first and second cohorts (cohort 1, winter; cohort 2, spring), respectively.

### Participant characteristics

Participants (*N* = 18) had an average age of 64 years (range 50–70), 11 women and 7 men, 10 self-identified as black and 8 white. About 44% reported smoking 11–20 cigarettes per day. Over 55% of participants reported an income of less than $20K per year and 79% were not working. Most participants in both cohorts had lung cancer (46%). There were differences in the two cohorts with regard to the active cancer treatment status at the time of intervention, 75% in cohort 1 and 20% in cohort 2 were undergoing cancer-directed therapy (Table 1).

### MBSC program feasibility and acceptability

**FIG. 2.**
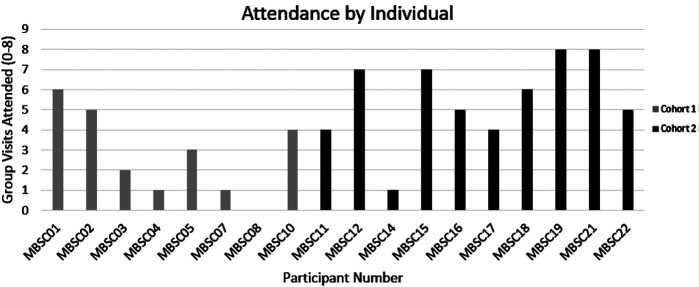
Mindfulness-based smoking cessation program attendance. MBSC, mindfulness-based smoking cessation.

Attendance during the first and second cohorts of the MBSC intervention was variable with three of eight participants completing four or more of the eight sessions in the first cohort. In the second cohort, 90% of participants attended four or more sessions (Fig. 2) and each week 70% of participants reported completing at least one home practice assignment. Acceptability and satisfaction with the intervention were notable across several themes that emerged from the focus groups. A total of 10 of the 18 enrolled participants attended 1 of the 2 focus groups (4 of 8 in cohort 1 and 6 of 10 in cohort 2). Focus group participants were similar in race and gender to participants enrolled in the program (seven women, three men, five African Americans, and five whites).

Participants were generally satisfied with the program. For example, participants remarked:

“I just wish it could go on. That's like us you know, we like the program and…we'd keep coming.”

“Yeah it takes me two and half hours to get here….I've enjoyed every day I've been here.”

Participants found the curriculum acceptable:

“Well, it's [MBSC Curriculum] our Bible now…it's ours to keep. If we do anything wrong, we can go back to the page and we read.”

They were satisfied with mindfulness training and other mind–body techniques:

“Yeah, I like the meditation. I did the yoga, but I preferred meditation because it's just more breathing and relaxation.”

“[I did] a lot of meditation. Sometimes I listen to it four times a day.”

MBSC groups were supportive:

“It's the support really. That's what I get…the support”

“…nobody in here judged me, which made me feel good about that. They encouraged me…”

Although participants were asked about components of the program that they would change or barriers to participation in the MBSC program, these did not emerge as dominant themes during the focus groups. Participants from the first cohort reported that winter weather and late time of day (4–6 pm) made attendance in the program less desirable due to traffic, the site not being as accessible in winter, and concern with safety walking to the parking garage in the dark.

### Smoking cessation

**FIG. 3.**
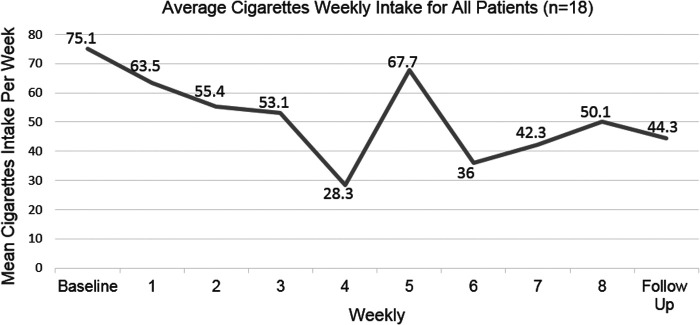
Mean weekly cigarette intake for all participants.

Overall, participants smoked fewer cigarettes after the intervention. One participant quit smoking for 14 days during the program and later relapsed. None of the participants continuously abstained from smoking. At baseline, mean weekly cigarette intake was 75.1 (SD = 52.8) cigarettes. During the last week of the intervention, mean weekly cigarette intake decreased to 50.1 (SD = 49.3) cigarettes (*p* = 0.131), and at the 3-month follow–up, there was a decrease to 44.3 cigarettes per week (*p* = 0.039) (Fig. 3).

Participants in the focus group reported that the MBSC program promoted smoking cessation:

“I always want a cigarette, but instead of running to satisfy that craving, think of something else for about 10 minutes or so and it's gone. That's what I've been doing.”

“This was good because it forced me to start programming myself against the act of smoking.”

“…I used to wake up in the morning and smell the smoke and that would be my queue to light me one up…now I sit around the house…not even worrying about cigarettes.”

“It gave me another way of thinking, another way of using my mind, my hands, the meditation…it was motivation.”

None of the participants sought any additional tobacco treatment counseling outside of the medical group visits through the telephone, web-based, or in-person hospital-based programs recommended in the curriculum. In addition, only one participant utilized recommended pharmacotherapy in the form of nicotine replacement therapy.

### Other positive lifestyle changes

Study participants reported additional positive lifestyle changes as a result of the program:

“…I've been walking so much more. And I know that has nothing to do with smoking, but I'm more aware of my health I think because of being in this program…”

“…I'm sleeping better at night. I'm thinking better…”

They also became more mindful and self-aware. For example:

“But we were more mindful, I was mindful of the fact that cut down, slow down, sleep more, cut it out, don't light it.”

“So my smell is coming back, my taste is back, because I've cut way down on cigarettes. I can taste my food, before I never tasted food.”

The program also improved interactions with others. One participant remarked:

“It's helped me deal with people better. I don't get so aggravated too quickly…”

## Discussion

The aim of this study was to assess the feasibility and acceptability of an MBSC medical group visit to engage racially diverse and low-socioeconomic cancer patients in evidence-based smoking cessation treatment. More than half of the participants in the MBSC program self-identified as black, had a low income, attended more than half of all the sessions, engaged in mindfulness and other integrative techniques, and smoked fewer cigarettes. Moreover, participants expressed an interest in continuing the program after completing the 8-week intervention.

The MBSC program was feasible. The authors observed moderately high recruitment and retention. Nearly one-third of all participants referred were enrolled in the MBSC program, and 90% of participants in the second cohort attended at least half of all the group visits. However, the attendance rate was lower in the first cohort. Many of the participants in cohort 1 were actively being treated for cancer, while the majority of participants in cohort 2 were cancer survivors. Participants undergoing active treatment were likely burdened with more clinic appointments and were likely too sick to participate. The attendance rate in the second cohort (70% completing five or more sessions) is comparable with a mindfulness-based group visit program for chronic pain conducted in a large, urban safety net hospital with 68% of enrolled participants attending five or more sessions.^23^

Although the findings from this study are based on a small cohort, the results are encouraging given the dearth of data on smoking cessation programs tailored specifically for low-SES and minority cancer patients. African American smokers with cancer are largely underrepresented in smoking cessation studies targeted for cancer patients and SES is generally not reported in these studies.^13–15,28–35^ Identifying strategies to feasibly recruit and engage racial/ethnic minority and low-SES cancer patients in smoking cessation programs was one of the main goals of this project. Recruitment strategies that likely enhanced referral and participation in the program included enlisting the support of medical assistants to collect smoking status on all patients in the clinic and providing study flyers to all active smokers. Strategies such as engaging medical assistants should be considered in other studies to improve recruitment of racial/ethnic minority and low-SES cancer patients.

Using a mindfulness-based approach in racially diverse and low-SES cancer patients to promote smoking cessation was acceptable to patients at Boston Medical Center, and this finding was consistent with other studies using mindfulness techniques in diverse and low-SES patient populations without cancer and in less diverse cancer populations.^22,23,36–38^ Qualitative data show that low-SES and minority cancer patients reported that the MBSC medical group visit provided a nonjudgmental and supportive environment for them to learn strategies to discuss their smoking habits and work through nicotine cravings and withdrawal. While the authors are unaware of any prior published studies assessing the impact of mindfulness training on smoking cessation in diverse and low-SES cancer patients, another study showed that a mindfulness-based addiction treatment program used for smoking cessation found that participants receiving mindfulness-based addiction treatment perceived a greater volitional control over smoking and reduced anxiety and cravings without smoking.^39^ Future studies should consider MBSC interventions in randomized control trials targeting low-SES and racial/ethnic minority cancer patients given the observed relationship of decreased cigarette intake after completion of the MBSC program.

Finally, despite a significant decrease in weekly cigarette intake, none of the participants quit smoking at the end of the intervention. However, evidence suggests that reducing the number of cigarettes smoked predicts future success with smoking cessation.^40,41^ Participants did not use telephone-based smoking cessation counseling through the Massachusetts quit line or any of the hospital-based smoking cessation counseling resources provided in the curriculum. Another study in a primary care population in an urban safety net hospital found similar findings.^42^ However, given the emerging data showing benefits of mindfulness training for smoking cessation, additional counseling may not be necessary, but mindfulness training alone may not be sufficient. Most participants in this study did not use the recommended pharmacotherapy. Current practice guidelines recommend the use of pharmacotherapy for successful smoking cessation, but underutilization of these medications by African Americans in particular has been previously reported.^43^ This MBSC program was not associated with use of evidence-based pharmacotherapy to assist with smoking cessation. Identifying other strategies to engage low-SES and minority cancer patients in the use of nicotine replacement therapy and other pharmacotherapies may improve quit rates.

While the findings provide valuable new insights regarding MBSC medical group visits in a racially diverse and low-SES population of cancer patients, there are several limitations to the study that warrant discussion. Given the pilot nature of the MBSC program, the sample size was small, there was no comparison group, and retention was variable. Participants in cohort 2 attended most of the group visits, which were conducted in late spring, and most participants were not undergoing active cancer treatment. Group visits for cohort 1 were conducted during winter, more participants were in active cancer treatment, and participation rates in the program were lower compared with cohort 2. Focus group participants in cohort 1 felt that the colder weather and time frame were barriers and another potential barrier is undergoing active cancer treatment. Another limitation was that the focus groups were limited to enrolled participants only. Lower attendance individuals, although similar in demographics to higher attendance individuals in the MBSC program, were less likely to participate in focus groups. These results on acceptability of the intervention may not be generalizable to lower attendance participants. Inviting patients who declined participation in the program may provide better insight on the acceptability of the program. Future studies on MBSC for cancer patients should consider clinical factors and time constraints unique to this population.

## Conclusion

Smoking cessation studies tailored for low-income and racial/ethnic minority cancer patients are limited. Findings from this pilot study demonstrate the feasibility and acceptability of MBSC medical group visits for low-SES and minority smokers with cancer. Future studies should consider the combination of a mindfulness-based medical group visit model with nicotine replacement therapy in a larger cohort using a randomized controlled design with longer follow-up to assess efficacy in improving smoking cessation rates in low-SES and minority cancer patient populations.
